# Regional variation in the potentially inappropriate first-line use of fluoroquinolones in Canada as a key to antibiotic stewardship? A drug utilization review study

**DOI:** 10.1186/s12879-021-06467-z

**Published:** 2021-08-03

**Authors:** Audray St-Jean, Dan Chateau, Matthew Dahl, Pierre Ernst, Nick Daneman, Ingrid S. Sketris, Jianguo Zhang, Fawziah Marra, Jacqueline Quail, Shawn Bugden, for the Canadian Network for Observational Drug Effect Studies (CNODES) Investigators

**Affiliations:** 1 Centre for Clinical Epidemiology, Lady Davis Institute, Jewish General Hospital, Montreal, QC Canada; 2 Manitoba Centre for Health Policy, Department of Community Health Sciences, Rady Faculty of Health Sciences, Max Rady College of Medicine, University of Manitoba, Winnipeg, MB Canada; 3 Institute for Clinical Evaluative Sciences, Toronto, ON Canada; 4 Division of Infectious Diseases, Sunnybrook Health Sciences Centre, Toronto, ON Canada; 5 College of Pharmacy, Dalhousie University, Halifax, NS Canada; 6 Department of Medicine, Cumming School of Medicine, University of Calgary, Calgary, AB Canada; 7 Faculty of Pharmaceutical Sciences, University of British Columbia, Vancouver, BC Canada; 8 Health Quality Council, Saskatoon, SK Canada; 9 Department of Community Health and Epidemiology, College of Medicine, University of Saskatchewan, Saskatoon, SK Canada; 10 School of Pharmacy, Health Sciences Centre, Memorial University of Newfoundland, 300 Prince Philip Drive, St John’s, NL A1B 3V6 Canada; 11 College of Pharmacy, Rady Faculty of Health Sciences, University of Manitoba, Winnipeg, MB Canada

**Keywords:** Antibiotics, Fluoroquinolones, Utilization

## Abstract

**Background:**

Serious adverse effects of fluoroquinolone antibiotics have been described for more than decade. Recently, several drug regulatory agencies have advised restricting their use in milder infections for which other treatments are available, given the potential for disabling and possibly persistent side effects. We aimed to describe variations in fluoroquinolone use for initial treatment of urinary tract infection (UTI), acute bacterial sinusitis (ABS), and acute exacerbation of chronic obstructive pulmonary disease (AECOPD) in the outpatient setting across Canada.

**Methods:**

Using administrative health data from six provinces, we identified ambulatory visits with a diagnosis of uncomplicated UTI, uncomplicated AECOPD or ABS. Antibiotic exposure was determined by the first antibiotic dispensed within 5 days of the visit.

**Results:**

We identified 4,303,144 uncomplicated UTI events among 2,170,027 women; the proportion of events treated with fluoroquinolones, mostly ciprofloxacin, varied across provinces, ranging from 18.6% (Saskatchewan) to 51.6% (Alberta). Among 3,467,678 ABS events (2,087,934 patients), between 2.2% (Nova Scotia) and 11.2% (Ontario) were dispensed a fluoroquinolone. For 1,319,128 AECOPD events among 598,347 patients, fluoroquinolones, mostly levofloxacin and moxifloxacin, ranged from 5.8% (Nova Scotia) to 35.6% (Ontario). The proportion of uncomplicated UTI and ABS events treated with fluoroquinolones declined over time, whereas it remained relatively stable for AECOPD.

**Conclusions:**

Fluoroquinolones were commonly used as first-line therapies for uncomplicated UTI and AECOPD. However, their use varied widely across provinces. Drug insurance formulary criteria and enforcement may be a key to facilitating better antibiotic stewardship and limiting potentially inappropriate first-line use of fluoroquinolones.

**Supplementary Information:**

The online version contains supplementary material available at 10.1186/s12879-021-06467-z.

## Background

Systemic oral fluoroquinolones are commonly prescribed antibiotics [1–4]. Given their advantageous pharmacokinetic and pharmacodynamics properties, such as high bioavailability and broad-spectrum antimicrobial activity [5], fluoroquinolones are among the most widely prescribed class of antibiotics. Some of this expanded use has been for milder infections, such as uncomplicated urinary tract infection (UTI), acute bacterial sinusitis (ABS), and uncomplicated acute exacerbation of chronic obstructive pulmonary disease (AECOPD), with limited evidence supporting their superiority to other first-line antibiotics [6–9]. Case reports and observational studies have indicated rare but severe adverse effects associated with fluoroquinolone use including tendon rupture [10], aortic aneurysm [11, 12], retinal detachment [13], and effects on the central and peripheral nervous system [14, 15]. Several safety warnings have been issued by regulatory agencies in the last decade. In 2016, the United States Food and Drug Administration (FDA) advised that the serious side effects of fluoroquinolone antibiotics generally outweigh their benefits in uncomplicated infections where other treatment alternatives are available [16]. In 2017 and 2018, Health Canada and the European Medicines Agency similarly recommended restricting fluoroquinolone use due to their disabling and potentially persistent side effects [17, 18].

Given the rare but potentially harmful adverse effects associated with fluoroquinolone antibiotic use, along with concerns of increasing fluoroquinolone resistance [19, 20], there is a need to ensure that they are prescribed for indications where there is a clear and proven benefit. Antibiotics resistance has important clinical and public health consequences and considerable associated cost impacts [21]. Using administrative health care databases from six Canadian provinces, we aimed to determine the proportion of initial antibiotic dispensations for uncomplicated UTI, ABS, and AECOPD in the outpatient setting across Canada, and to describe variations in the use of systemic oral fluoroquinolones.

## Methods

### Study design and population

This study was conducted by the Canadian Network for Observational Drug Effect Studies (CNODES) [22, 23]. We formed three retrospective population-based cohorts, one for each infection type, using administrative health care data from six Canadian provinces (Alberta, British Columbia, Manitoba, Nova Scotia, Ontario, and Saskatchewan) between January 1, 2005 and March 31, 2017 (range dependent on data availability at each site). Site-specific study periods were reported in Additional file 1: Figures S1, S2, and D3. Briefly, the databases include population-level data on physician billings, hospitalization data, and prescription drug claims. Due to prescription drug claims data availability, analyses were limited to those aged 18 and older in Alberta, and those aged 65 and older in Nova Scotia and Ontario. Prescription drug data is available for all ages in the other provinces. A common protocol was implemented separately at each participating site. The study protocol was approved by the institutional review boards at all participating sites. All study protocols were carried out in accordance with relevant guidelines and regulations at each participating site.

### Study cohorts

Within each province, we identified ambulatory visits with a diagnosis for UTI (ICD-9-CM: 595.x, 599.x; ICD-10-CA: N30.x, N39.x), ABS (ICD-9-CM: 461.x; ICD-10-CA: J01.x) or COPD (ICD-9-CM: 490.x, 491.x, 492.x, 496.x; ICD-10-CA: J40.x-J44.x). Cohort entry date was defined by the visit date. Antibiotic exposure was determined by the first antibiotic dispensation (oral systemic fluoroquinolone or other oral antibiotic) occurring within ± 5 days of the event date. Exposure was defined using the Anatomical Therapeutic Chemical (ATC) codes J01M for oral fluoroquinolones (including but not limited to ciprofloxacin, levofloxacin, moxifloxacin, norfloxacin and ofloxacin) and J01 (excluding J01M) for other oral antibiotics. Patients were eligible to enter the study cohorts multiple times with each new event.

#### Uncomplicated UTI

Patients with recurrent UTI based on an event in the prior 90 days, or those with a hospitalization in the prior 30 days were excluded from the UTI cohort. We excluded males and patients with a diagnosis suggesting a complicated UTI in the year prior to cohort entry. These diagnoses included structural abnormality of urinary tract (including stones), ureteral abnormalities, vesicoureteral reflux, neurogenic bladder, neurologic conditions, diabetes or pregnancy (in the 270 days prior to the UTI event date). Patients were also required to have at least 365 days of health care coverage prior to the UTI event and at least 5 days of coverage after the event.

#### Acute bacterial sinusitis

For the ABS cohort, patients with a sinusitis event or hospitalization in the preceding 30 days were excluded. We also excluded patients with less than 365 days of health care coverage prior to the ABS event and those with less than 5 days of coverage after the event.

#### Acute exacerbation of COPD

Patients aged less than 66 years old were excluded from the AECOPD cohort. To limit the cohort to uncomplicated AECOPD, patients with an event, hospitalization, or use of antibiotics or oral corticosteroids in the 90 days prior to cohort entry were excluded. We excluded patients with a history of heart failure or ischemic heart disease in the year prior. Patients were also required to have at least 365 days of health care coverage prior to the AECOPD event and at least 5 days of coverage after the event.

### Review of provincial formularies

We conducted a review of public drug insurance formulary criteria for systemic oral fluoroquinolones in each province. Criteria for ciprofloxacin, levofloxacin, moxifloxacin, and norfloxacin were assessed in October 2016 through the National Prescription Drug Utilization Information System (NPDUIS) Database developed by the Canadian Institute of Health Information (CIHI) [24]. Current versions of the provincial drug plans are accessible online from the respective health ministries [25–30]. Each fluoroquinolone was categorized by their benefit status: general benefit (no specific requirement for reimbursement), limited benefit (restricted to specific criteria, for example requiring a particular diagnosis or a special authorization for reimbursement), or non-benefit.

### Statistical analysis

The proportion of events initially treated with a fluoroquinolone was estimated by calculating the percentage of fluoroquinolone dispensations among all antibiotic dispensations within a year. The overall fluoroquinolone use represented the mean of all data aggregated for years where data is available in at least two provinces, i.e. from 2005 to 2015. The overall trend in use over the study period was evaluated using linear regression. The change in fluoroquinolone dispensations per year was expressed as the beta coefficient and its corresponding 95% confidence intervals (CIs). Results were presented by province and by calendar year.

## Results

### Uncomplicated UTI

**Fig. 1:**
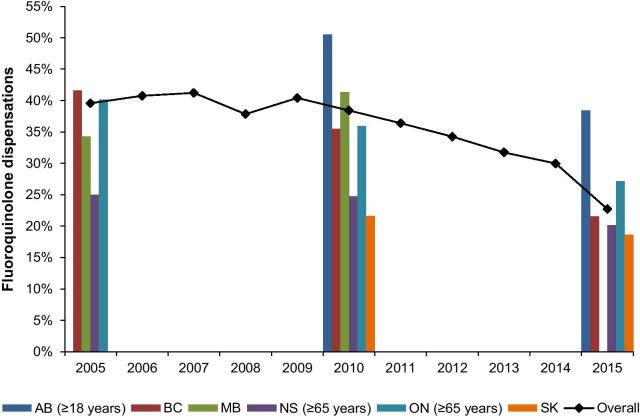
Proportion of initial fluoroquinolone dispensations for uncomplicated UTI between 2005 and 2015 in Canadian provinces*. *Data are presented as percentage of fluoroquinolone dispensations by province for years 2005, 2010 and 2015, and overall fluoroquinolone use is represented as the mean of all data aggregated for years where data is available in at least two provinces. Data not available for AB (2005–2008), MB (2015) and SK (2005–2007). *AB* Alberta, *BC* British Columbia, *MB* Manitoba, *NS* Nova Scotia, *ON* Ontario, *UTI* urinary tract infection, *SK* Saskatchewan

We identified 4,303,144 visits for uncomplicated UTI among 2,170,027 women (Additional file 1: Figure S1). Of these, 67.6% were treated with an antibiotic. The use of fluoroquinolones varied significantly across provinces, ranging from 18.6% in Saskatchewan to 51.6% in Alberta (Fig. 1). Overall, the proportion of antibiotic-treated uncomplicated UTI events treated with a fluoroquinolone declined over time (Fig. 1). We observed a 1.5% (95% CI: 0.9 to 2.1) decrease in fluoroquinolone dispensations per year.

The three antibiotics most commonly prescribed for incident uncomplicated UTIs remained similar over time (Table 1). Nitrofurantoin was the most commonly dispensed antibiotic in all provinces except Manitoba, where it was the third most commonly dispensed antibiotic at the start and end of the study period. Ciprofloxacin and trimethoprim/sulfamethoxazole were the two other commonly dispensed antibiotics. Among fluoroquinolones, ciprofloxacin was by far the most commonly dispensed drug followed by norfloxacin. In all provinces, norfloxacin use declined over time whereas ciprofloxacin use increased or remained relatively stable (data not shown).

**Table 1:** Top 3 most common antibiotic dispensations associated with uncomplicated UTI, ABS and AECOPD events

Province	Year	Antibiotic dispensations		
		1	2	3
*Uncomplicated UTI*				
Alberta	2009	**Ciprofloxacin**	Nitrofurantoin	tmp/smx
	2015	Nitrofurantoin	**Ciprofloxacin**	tmp/smx
British Columbia	2005	**Ciprofloxacin**	tmp/smx	nitrofurantoin
	2015	Nitrofurantoin	**Ciprofloxacin**	tmp/smx
Manitoba	2005	tmp/smx	**Ciprofloxacin**	Nitrofurantoin
	2014	**Ciprofloxacin**	tmp/smx	Nitrofurantoin
Nova Scotia	2005	tmp/smx	Nitrofurantoin	**Ciprofloxacin**
	2015	Nitrofurantoin	tmp/smx	**Ciprofloxacin**
Ontario	2005	Nitrofurantoin	**Norfloxacin**	tmp/smx
	2015	Nitrofurantoin	**ciprofloxacin**	tmp/smx
Saskatchewan	2008	Nitrofurantoin	tmp/smx	**Ciprofloxacin**
	2015	Nitrofurantoin	tmp/smx	**Ciprofloxacin**
*ABS*				
Alberta	2009	Amoxicillin	Clarithromycin	Azithromycin
	2015	Amoxicillin	Clarithromycin	amox/clav
British Columbia	2005	Amoxicillin	Clarithromycin	Azithromycin
	2015	Amoxicillin	Clarithromycin	amox/clav
Manitoba	2005	Amoxicillin	Azithromycin	Clarithromycin
	2014	Amoxicillin	Azithromycin	Clarithromycin
Nova Scotia	2005	Amoxicillin	Cefuroxime	Azithromycin
	2015	Amoxicillin	amox/clav	Cefuroxime
Ontario	2005	Amoxicillin	Clarithromycin	Azithromycin
	2016	Amoxicillin	Azithromycin	amox/clav
Saskatchewan	2008	Amoxicillin	Azithromycin	Cephalexin
	2015	Amoxicillin	Azithromycin	Clarithromycin
*AECOPD*				
Alberta	2009	Clarithromycin	**Levofloxacin**	Azithromycin
	2015	**Levofloxacin**	Azithromycin	Doxycycline
British Columbia	2005	Clarithromycin	Azithromycin	Amoxicillin
	2015	Clarithromycin	Doxycycline	Amoxicillin
Manitoba	2005	Amoxicillin	Azithromycin	Clarithromycin
	2014	Azithromycin	Amoxicillin	**Levofloxacin**
Nova Scotia	2005	Azithromycin	Amoxicillin	Cefuroxime
	2015	Doxycycline	Amoxicillin	Clarithromycin
Ontario	2005	Clarithromycin	Azithromycin	**Levofloxacin**
	2017	Azithromycin	Amoxicillin	amox/clav
Saskatchewan	2008	Doxycycline	Azithromycin	Amoxicillin
	2015	Azithromycin	Doxycycline	Amoxicillin

Data are presented by province at the start and end of study period. Fluoroquinolone antibiotics are in bold

*ABS* acute bacterial sinusitis, *AECOPD* acute exacerbation of chronic obstructive pulmonary disease, *amox/clav* amoxicillin/clavulanic acid, *tmp/smx* trimethoprim/sulfamethoxazole, *UTI* urinary tract infection

### Acute bacterial sinusitis

**Fig. 2:**
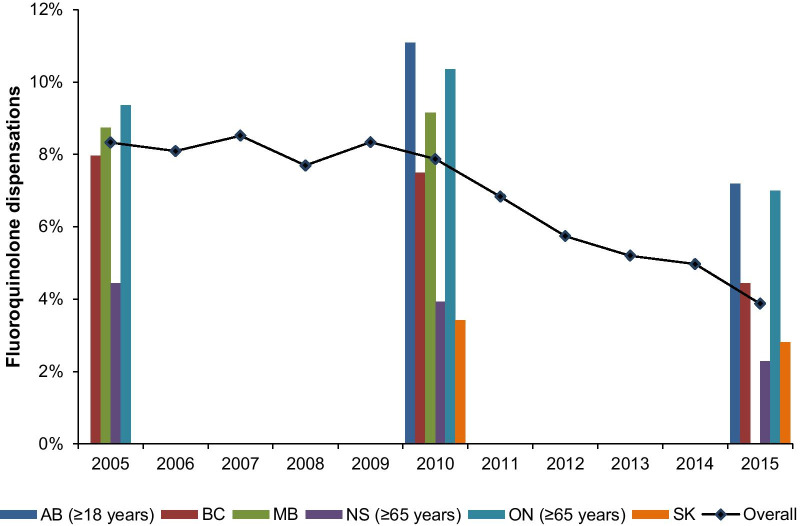
Proportion of initial fluoroquinolone dispensations for acute bacterial sinusitis between 2005 and 2015 in Canadian provinces*. *Data are presented as percentage of fluoroquinolone dispensations by province for years 2005, 2010 and 2015, and overall fluoroquinolone use is represented as the mean of all data aggregated for years where data is available in at least two provinces. Data not available for AB (2005–2008), MB (2015) and SK (2005–2007). *AB* Alberta, *BC* British Columbia, *MB* Manitoba, *NS* Nova Scotia, *ON* Ontario, *UTI* urinary tract infection, *SK* Saskatchewan

Over the study period, we identified 3,467,678 ABS events among 2,087,934 unique individuals (Additional file 1: Figure S2). The proportion of ABS events treated with an antibiotic was 92.9% overall and fluoroquinolones were not commonly used, representing 2.2% (Nova Scotia) to 11.2% (Ontario) of initial antibiotic dispensations (Fig. 2). We observed an overall decline in fluoroquinolone use, with dispensations decreasing of 0.45% (95% CI: 0.31 to 0.60) per year.

The three antibiotics most commonly dispensed for ABS remained relatively similar over the study period (Table 1). Amoxicillin was the most commonly dispensed antibiotic in all provinces, followed by the macrolides (azithromycin/clarithromycin) and amoxicillin/clavulanic acid. In all provinces except Nova Scotia, moxifloxacin was the most commonly dispensed fluoroquinolone, followed by levofloxacin and ciprofloxacin (data not shown). In Nova Scotia, ciprofloxacin was more commonly dispensed than moxifloxacin.

### Acute exacerbation of COPD

**Fig. 3:**
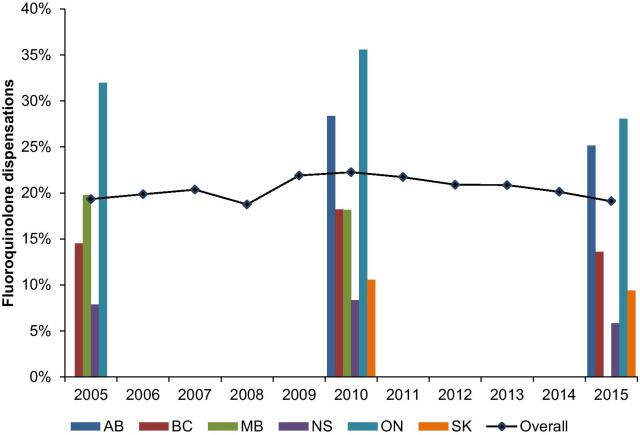
Proportion of initial fluoroquinolone dispensations for acute exacerbation of COPD between 2005 and 2015 in Canadian provinces*. *Data are presented as percentage of fluoroquinolone dispensations by province for years 2005, 2010 and 2015, and overall fluoroquinolone use is represented as the mean of all data aggregated for years where data is available in at least two provinces. Data not available for AB (2005–2008), MB (2015) and SK (2005–2007). *AB* Alberta, *BC* British Columbia, *MB* Manitoba, *NS* Nova Scotia, *ON* Ontario, *UTI* urinary tract infection, *SK* Saskatchewan

Among 598,347 unique individuals aged 66 and older, 1,319,128 AECOPD events were identified (Additional file 1: Figure S3). Of these, 20.5% were treated with an antibiotic. Among events treated with an antibiotic, the use of fluoroquinolones in the first-line treatment of AECOPD varied by province, ranging from 5.8% in Nova Scotia to 35.6% in Ontario (Fig. 3). The overall use of fluoroquinolones was relatively stable over time. The change in fluoroquinolone dispensations per year was not significant (0.05% [95% CI: − 0.22 to 0.31]).

Over the study period, the three most commonly dispensed antibiotics for AECOPD remained relatively similar in all provinces (Table 1). The most commonly dispensed antibiotics were the macrolides (azithromycin/clarithromycin), doxycycline and amoxicillin. In all provinces, moxifloxacin and levofloxacin were the most commonly dispensed fluoroquinolones followed by ciprofloxacin (data not shown).

### Review of provincial drug formularies

A summary of provincial formularies for fluoroquinolones in 2016 by their benefit status (general benefit, limited, non-benefit) is presented in Table 2. British Columbia was the only province where fluoroquinolones were listed as general benefit, and where levofloxacin was not listed on the provincial drug plan. Manitoba, Nova Scotia, and Saskatchewan were the provinces with the higher number of formulary restrictions for fluoroquinolones. There was no formulary restriction for the use of norfloxacin in Alberta, British Columbia, and Ontario.

**Table 2:** Summary of Canadian provincial formularies for oral fluoroquinolone antibiotics in 2016

	AB	BC	MB	NS	ON	SK
Ciprofloxacin	L	B	L	L	L	L
Levofloxacin	L	NB	L	L	L	L
Moxifloxacin	L	B	L	L	L	L
Norfloxacin	B	B	L	L	B	L

Information assessed in October 2016 through the National Prescription Drug Utilization Information System (NPDUIS) Database. Current versions of the provincial drug plans are accessible online from the respective health ministries

B (benefit): no specific requirements for reimbursement

L (limited): restricted to specific criteria

NB (non-benefit): not available through the public drug plan

*AB* Alberta, *BC* British Columbia, *MB* Manitoba, *NS* Nova Scotia, *ON* Ontario, *SK* Saskatchewan

## Discussion

In our retrospective cohorts, we observed that systemic oral fluoroquinolones were commonly used in the first-line treatment of uncomplicated UTI and AECOPD in Canada. However, the proportion of fluoroquinolone dispensations varied widely across provinces. Fluoroquinolones were infrequently used in the first-line treatment of ABS. We noted a trend towards decreasing use of fluoroquinolones for uncomplicated UTI and ABS between 2005 and 2015.

We observed potentially inappropriate first-line use of systemic oral fluoroquinolones in the treatment of uncomplicated UTI and AECOPD. Fluoroquinolones, primarily ciprofloxacin, were frequently dispensed in the first-line treatment of uncomplicated UTI. However, the use of fluoroquinolones for this indication tended to decrease in all provinces during the study period, which is consistent with guideline recommendations to restrict fluoroquinolone use to second-line in women with uncomplicated UTI [7, 31]. Respiratory fluoroquinolones, levofloxacin and moxifloxacin, were commonly prescribed for AECOPD events treated with antibiotics although their use is recommended for patients with specific risk factors or treatment failure with first-line antibiotics [32]. The proportion of AECOPD events treated with a fluoroquinolone remained relatively stable over time. A relatively small proportion of ABS events were treated with fluoroquinolones but guidelines suggest that they should be used in second-line only [33, 34]. However, we noted that a substantial proportion of ABS events were treated with antibiotics in our study cohort, although the recommendations suggest limiting their use to patients with severe symptoms or failing to respond to intranasal corticosteroids after 72 h [33]. Additionally, the majority of acute sinusitis cases are of viral etiology, with only 0.5 to 2% progressing to ABS [35]. We observed a trend towards decreasing fluoroquinolone use for this indication. Our findings of fluoroquinolone use for these three infections and overall decline in use of this class of antibiotic have also been previously reported in Canada and in the United States [1, 36–39].

Differences in provincial formulary criteria and enforcement, local practice, antibiotic resistance rates, and marketing patterns may partly explain the large interprovincial variations observed in the use of fluoroquinolones. As each province and territory has its own publicly funded drug plan, differences in the coverage of drugs are expected. A previous review of provincial drug formulary for antimicrobials has shown that in comparison with other antimicrobials, fluoroquinolones are a class with more restricted benefits [4]. From a review of provincial formularies for fluoroquinolones in 2016, we observed that coverage of fluoroquinolones varies across provinces. Fluoroquinolones were more restricted in Manitoba, while British Columbia was the only province with no restrictions for this class, although levofloxacin was not listed as a benefit. Our results showed that fluoroquinolone dispensations tended to be lower in Nova Scotia and Saskatchewan compared to other provinces. Although Manitoba, Nova Scotia, and Saskatchewan, have a similar restricted benefits for fluoroquinolones, their utilization differs, which may be explained by enforcement and management of formulary restrictions, such as the use of criteria codes on prescription or written forms [4]. In general, prescribing rates are expected to be lower in provinces with a greater number of formulary restrictions [4] and studies have described a reduction in the use of fluoroquinolones following implementation of specific restrictions [40, 41]. We also noted variations in the specific criteria for coverage of ciprofloxacin, levofloxacin, and moxifloxacin across provinces. For example, ciprofloxacin is specifically indicated for the treatment of genitourinary tract infections in Alberta and Ontario which could potentially explain the higher proportion of fluoroquinolone dispensations observed in these provinces for uncomplicated UTI. Local practice patterns could also explain some of these variations in the use of fluoroquinolones. For example, Alberta [42], British Columbia [43], Nova Scotia [44], and Saskatchewan [45] have had educational programs that may have influenced antibiotic prescribing. A recent survey of primary health care providers indicated that fluoroquinolone-prescribing habits were similar for uncomplicated cystitis, uncomplicated pyelonephritis, acute bacterial exacerbation of chronic bronchitis in COPD and ABS across Canada [46]. Other factors such as variations in antibiotic-resistance, adherence to treatment guidelines or marketing patterns, across jurisdictions may also contribute to the interprovincial differences observed in the use of fluoroquinolones. Lastly, an additional explanation is the heterogeneity in the prescription drug data available across the different study sites, i.e. all vs. government reimbursed dispensations. All dispensations (including those for which patient pay out-of-pocket) are captured in Alberta, British Columbia, Manitoba, and Saskatchewan, whereas only provincial government reimbursed dispensations are captured in Nova Scotia and Ontario.

Our study has limitations. Our data is limited to antibiotics dispensed in outpatient pharmacies and thus cannot be generalized to other settings of care. Inter-provincial comparisons in UTI and ABS treatment must take into account the fact that some provinces (such as Nova Scotia and Ontario) only have drug dispensation data available for older adults ≥ 65 years old. Also, not all provinces were represented in our sample and data was not available for all years for the represented provinces. Event definitions are based on outpatient diagnosis codes and do not include clinical characteristics or laboratory values. Although antibiotic exposure was defined as the first antibiotic dispensed within 5 days of the event, we could not be certain the antibiotic was actually prescribed for the indication listed as the diagnosis for the physician visit. While we were able to document provincial formulary prescribing criteria for 2016, these may have varied over the study period and do not consider any supplementary private drug insurance restrictions. We were unable to document all influences on prescribing such as continuing professional development, academic detailing, and antibiotic stewardships programs. Lastly, our findings only provide a descriptive snapshot of fluoroquinolone use in uncomplicated UTI, ABS, and AECOPD in Canada. Utilization data was also used by the FDA during the safety review of fluoroquinolones to guide the policy decision around the 2016 warning. Further studies are needed to evaluate the outcomes of fluoroquinolone therapy compared to first-line antibiotics and assess their need in more complicated situations [47, 48].

## Conclusions

In summary, systemic oral fluoroquinolones were commonly used as first-line therapies in Canada, particularly for uncomplicated UTI and AECOPD. However, first-line fluoroquinolone use varied widely across provinces. There was a decline in the proportion of uncomplicated UTI and ABS events treated with fluoroquinolones between 2005 and 2015. Drug formulary criteria and enforcement in addition to prescriber and public education are several key approaches to promoting better antibiotic stewardship and limiting potentially inappropriate first-line use of fluoroquinolones.

## Supplementary Information

Below is the link to the electronic supplementary material.

**Additional file 1: Figure S1.** Flow diagram of study cohort creation for uncomplicated UTI. **Figure S2.** Flow diagram of study cohort creation for ABS. **Figure S3.** Flow diagram of study cohort creation for AECOPD.

## Data Availability

The data that support the findings of this study are not publicly available, in accordance with site-specific privacy restrictions. The data that support the findings of this study are available, with submission of appropriate ethics and data access approvals, from Alberta Health, the British Columbia Ministry of Health, the Manitoba Centre for Health Policy, the Health Data Nova Scotia (HDNS), the Institute for Clinical Evaluative Sciences (ICES) in Ontario, and the Saskatchewan Health Quality Council.

## Abbreviations

ABS: Acute bacterial sinusitis
AECOPD: Acute exacerbation of chronic obstructive pulmonary disease
CIHI: Canadian Institute of Health Information
CI: Confidence intervals
CNODES: Canadian Network for Observational Drug Effect Studies
NPDUIS: National Prescription Drug Utilization Information System
FDA: Food and Drug Administration
UTI: Urinary tract infection
