# Supplementary material for: Regional variation in the potentially inappropriate first-line use of fluoroquinolones in Canada as a key to antibiotic stewardship? A drug utilization review study
Source: BMC Infect Dis. 2021 Aug 3;21:733. doi: 10.1186/s12879-021-06467-z (PMC8330086; doi:10.1186/s12879-021-06467-z)
Supplement: Supplementary file 1 — Additional file 1: Figure S1. Flow diagram of study cohort creation for uncomplicated UTI. Figure S2. Flow diagram of study cohort creation for ABS. Figure S3. Flow diagram of study cohort creation for AECOPD. [file 12879_2021_6467_MOESM1_ESM.docx]

**Additional material**

UTI diagnoses events between January 1, 2005 and latest date of data available for the site^*^

(*n* = 13,372,202)

**Exclusions** (*n* = 9,069,058)

- UTI event in the prior 90 days

(*n* = 5,369,325)

- UTI event occurs within 5 days prior to latest date of data available for the site

(*n* = 718,560)

- <365 days of health coverage prior to the UTI event date (*n* = 120,689)
- No health coverage for at least 5 days after the UTI event date (*n* = 9,544)
- Hospitalization in the prior 30 days

(*n* = 315,772)

- Male or missing sex (*n* = 1,649,935)
- Diagnosis of a condition indicating UTI is complicated (*n* = 885,233)^†^

Number of new uncomplicated UTI events

(*n* = 4,303,144)

Number of unique individuals

(*n* = 2,170,027)

# Figure S1: Flow diagram of study cohort creation for uncomplicated UTI

*Study period varied across provinces: Alberta (January 1, 2009 to March 31, 2015), British Columbia (January 1, 2005 to December 31, 2015), Manitoba (January 1, 2005 to December 31, 2014), Nova Scotia (January 1, 2005 to March 31, 2015), Ontario (January 1, 2005 to March 31, 2015), and Saskatchewan (January 1, 2008 to March 31, 2016).

^†^Complicated UTI defined by a diagnosis of structural abnormality of urinary tract (including stones), ureteral abnormalities, vesicoureteral reflux, neurogenic bladder, neurologic conditions, or diabetes in the year prior to the event date, or pregnancy in the 270 days prior.

Note: Patients were allowed to enter the cohort multiple times with a new uncomplicated UTI event.

ABS diagnoses events between January 1, 2005 and latest date of data available for the site^*^

(*n* = 4,426,413)

**Exclusions** (*n* = 958,735)

- Sinusitis event in the prior 30 days

(*n* = 590,709)

- Hospitalization in the prior 30 days

(*n* = 39,082)

- Sinusitis event occurs within 5 days prior to latest date of data available for the site

(*n* = 276,449)

- <365 days of health coverage prior to the sinusitis event date (*n* = 52,314)
- No health coverage for at least 5 days after the sinusitis event date (*n* = 181)

Number of new ABS events

(*n* = 3,467,678)

Number of unique individuals

(*n* = 2,087,934)

# Figure S2: Flow diagram of study cohort creation for ABS

*Study period varied across provinces: Alberta (January 1, 2009 to March 31, 2015), British Columbia (January 1, 2005 to December 31, 2015), Manitoba (January 1, 2005 to December 31, 2014), Nova Scotia (January 1, 2005 to March 31, 2015), Ontario (January 1, 2005 to March 31, 2016), and Saskatchewan (January 1, 2008 to March 31, 2016).

Note: Patients were allowed to enter the cohort multiple times with a new ABS event.

AECOPD diagnoses events between

January 1, 2005 and latest date of data

available for the site^*^

(*n* = 12,166,600)

**Exclusions** (*n* = 10,847,472)

- <66 years on the AECOPD event date

(*n* = 5,357,087)

- AECOPD event in the prior 90 days

(*n* = 4,032,399)

- Hospitalization in the prior 90 days

(*n* = 302,034)

- Antibiotic use in the prior 90 days

(*n* = 587,415)

- Oral corticosteroid use in the prior 90 days

(*n* = 68,343)

- AECOPD event occurs within 5 days prior to latest date of data available for the site

(*n* = 41,983)

- <365 days of health coverage prior to the AECOPD event date (*n* = 8,119)
- History of heart failure or ischemic heart disease in the year prior to or on the AECOPD event date (*n* = 449,494)
- No health coverage for at least 5 days after the AECOPD event date (*n* = 598)

Number of new AECOPD events

(*n* = 1,319,128)

Number of unique individuals

(*n* = 598,347)

# Figure S3: Flow diagram of study cohort creation for AECOPD

*Study period varied across provinces: Alberta (January 1, 2009 to March 31, 2015), British Columbia (January 1, 2005 to December 31, 2015), Manitoba (January 1, 2005 to December 31, 2014), Nova Scotia (January 1, 2005 to March 31, 2015), Ontario (January 1, 2005 to March 31, 2017), and Saskatchewan (January 1, 2008 to March 31, 2016).

Note: Patients were allowed to enter the cohort multiple times with a new AECOPD event.
